# What Should Our Policy Be?

**Published:** 1914-08-29

**Authors:** 


### HOSPITALS AND THEIR CONTRACTS.

### What Should Our Policy be?

### A PROVINCIAL SECRETARY'S VIEW.

Many specious excuses are being urged for the cancellation of contracts on account of the war, but there seems no doubt that, so far as the legality of the contracts is concerned, committees are justified in holding contractors to their bargains.

Some of the excuses given by contractors for failure to supply or in justification of enhanced prices are really ludicrous were it not that they are made apparently in all seriousness. A firm, for example, writes to my institution, which possesses over 250 beds: "Since the declaration of war the Admiralty orders have been an enormous drain on our stocks, and hospitals and other institutions throughout the country whose contracts we hold have also ordered largely, and our raw materials cost us double. . . ." In other words, the firm has been executing Government orders at prices doubtless remunerative from stocks which they had contracted to supply to hospitals, and they therefore ask these hospitals to pay double! Was ever such a feeble reason submitted or a weaker case presented? Surely an apology to the hospitals would be nearer the mark than an application for increased prices.

Again, it is most unlikely that tendering houses are not themselves covered to the extent of their contracts, and therefore there is ample reason for hospitals taking up a strict legal attitude. Point was recently made with regard to a building contract. A contractor having once bound himself to a price would not ask for reconsideration because of the fact, for example, that during the operations the rates of wages of one or two sections of his men had been advanced and he could not reasonably have expected, when giving in his price, to have contemplated such an emergency. The further one goes the more justified appear the reasons for committees holding contractors to their bargains.

It would be infinitely to the credit of contractors if they in general follow the lead of a well-known surgical dressings house not to ask for alterations in contract prices, in the hope that by the date contracts expire prices will have reassumed normal conditions. Such a decision would show generous appreciation of the position, and firms who follow it might rely upon requisitions being kept down to the lowest limit.

